# Secondary metabolites from plant‐associated *Pseudomonas* are overproduced in biofilm

**DOI:** 10.1111/1751-7915.13598

**Published:** 2020-08-09

**Authors:** Laura Rieusset, Marjolaine Rey, Daniel Muller, Jordan Vacheron, Florence Gerin, Audrey Dubost, Gilles Comte, Claire Prigent‐Combaret

**Affiliations:** ^1^ CNRS UMR‐5557 INRAe UMR‐1418 Ecologie Microbienne VetAgroSup Université de Lyon Université Claude Bernard Lyon1 43 Boulevard du 11 novembre 1918 Villeurbanne 69622 France; ^2^ Department of Fundamental Microbiology University of Lausanne Lausanne 1015 Switzerland

## Abstract

Plant rhizosphere soil houses complex microbial communities in which microorganisms are often involved in intraspecies as well as interspecies and inter‐kingdom signalling networks. Some members of these networks can improve plant health thanks to an important diversity of bioactive secondary metabolites. In this competitive environment, the ability to form biofilms may provide major advantages to microorganisms. With the aim of highlighting the impact of bacterial lifestyle on secondary metabolites production, we performed a metabolomic analysis on four fluorescent *Pseudomonas* strains cultivated in planktonic and biofilm colony conditions. The untargeted metabolomic analysis led to the detection of hundreds of secondary metabolites in culture extracts. Comparison between biofilm and planktonic conditions showed that bacterial lifestyle is a key factor influencing *Pseudomonas* metabolome. More than 50% of the detected metabolites were differentially produced according to planktonic or biofilm lifestyles, with the four *Pseudomonas* strains overproducing several secondary metabolites in biofilm conditions. In parallel, metabolomic analysis associated with genomic prediction and a molecular networking approach enabled us to evaluate the impact of bacterial lifestyle on chemically identified secondary metabolites, more precisely involved in microbial interactions and plant‐growth promotion. Notably, this work highlights the major effect of biofilm lifestyle on acyl‐homoserine lactone and phenazine production *in P. chlororaphis* strains.

## Graphical abstract

*Pseudomonas* strains overproduce a higher diversity of secondary metabolites in biofilm colony than in planktonic lifestyle. In particular, the biosynthesis of key compounds implicated in bacterial biotic interactions like AHLs or phenazines are strongly enhanced in biofilms. Biofilms are thus key ways to enhance the biocontrol and biostimulant activities of bioinoculant for agriculture purposes.

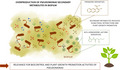

## Introduction

Plant roots exudate in the rhizosphere, the soil area under root influence, a wide diversity of chemical compounds (Haichar *et al*., 2014). Most of these compounds attract microorganisms and promote their growth (Danhorn and Fuqua, 2007). The microbial community associated to the plant roots is known as the rhizo‐microbiota (Brink, 2016). Within this rhizo‐microbiota, some microorganisms can promote plant growth and protect plants against pathogens, thanks to several indirect or direct mechanisms (Vacheron *et al*., 2013). To express their plant‐beneficial properties, bacteria have to be competitive and able to colonize the surface of plant tissues (Vacheron *et al*., 2013). They are able to form biofilms on the plant roots inside the rhizosphere, as well as on soil particles, or on fungal mycelium (Ramey *et al*., 2004). The ability to form biofilm is an advantage for microorganisms to be competitive in the rhizosphere (Pandin *et al*., 2017). Biofilms can be described as aggregates of microorganisms, generally embedded in an extracellular matrix and adhering to living or inert surfaces. This sessile mode constitutes the preferential lifestyle of microorganisms (Prigent‐Combaret *et al*., 2012). Biofilm formation is a dynamic process and relies on multiple biotic and abiotic factors such as nutrient availability, secretion of extracellular material and social competition (Flemming *et al*., 2016). By providing a micro‐environment where the diffusion of compounds is limited within the matrix, the biofilm facilitates intercellular signalling. Given the properties of the extracellular matrix and the facilitated interactions between the microorganisms, the biofilm lifestyle is clearly different from the lifestyle of planktonic cells (Prigent‐Combaret *et al*., 1999). New properties can emerge in biofilms that cannot be predictable from studies on free‐living organisms (Pandin *et al*., 2017). In the rhizosphere, biofilm lifestyle plays a key role in plant–bacteria and bacteria–bacteria interactions and triggers the expression of plant‐beneficial properties by beneficial bacteria (Al‐Ali *et al*., 2017; Besset‐Manzoni *et al*., 2018).

The fluorescent *Pseudomonas* group regroups a wide diversity of rhizosphere plant‐beneficial bacteria that can colonize the rhizosphere of different plant species and form biofilm (Noirot‐Gros *et al*., 2018). They can interact with various host plants and protect them against pathogens thanks to the biosynthesis of antimicrobial products like 2,4‐diacetylphloroglucinol (DAPG), phenazines, pyrrolnitrin or alkylresorcinol (Couillerot *et al*., 2009; Gross and Loper, 2009; Vacheron *et al*., 2013). They also promote plant growth through the modulation of plant hormonal pathways (Brazelton *et al*., 2008). Genome mining allows to predict the ability of strains to produce bioactive secondary metabolites (Loper *et al*., 2012; Oni *et al*., 2015). But genome mining does not take into account the biotic or abiotic factors that can influence the rhizobacterial production of secondary metabolites. Indeed, metabolites constitute the final product of gene expression and their production may depend on a myriad of external factors (Wolfender *et al*., 2013). Innovation in analytical chemistry and the development of metabolomics enable to study large sets of metabolites actually produced by microorganisms in biological samples, making it possible to correlate metabolite production to microbial physiology (Favre *et al*., 2017).

In this study, we applied a metabolomics approach to evaluate if part of the metabolome of *Pseudomonas* strains differs according to biofilm or planktonic lifestyle. Metabolome of a given strain is defined as all metabolites produced by this strain at a given time. Here, regarding our analytical workflow, our study focused on secondary metabolites with molecular masses lower than 1000 Da, but may also include some primary metabolites. *Pseudomomas* strains considered in this study were *P. koreensis* JV222, *P. chlororaphis* JV395B and *P. chlororaphis* JV497 isolated from soil or maize roots as well as *P. kilonensis* F113 isolated from sugar beet rhizosphere (Shanahan *et al*., 1992; Vacheron *et al*., 2016). The metabolomics approach developed in this study allows us to (i) detect compounds predicted by genome mining in the 4 *Pseudomonas* genomes and to (ii) highlight the important diversity of unknown compounds produced by *Pseudomonas* strains. Our results showed that bacterial lifestyle constitutes a key factor that influences the production of secondary metabolites in the four studied *Pseudomonas* strains. We found that more than 50% of the detected compounds are differentially produced according to the lifestyle of the bacteria and that the majority of *Pseudomonas* secondary metabolites are overproduced in biofilm colony. Uncharacterized compounds as well as secondary metabolites involved in bacterial biotic interactions like acyl‐homoserine lactones (AHL) or phenazines are strongly influenced by biofilm lifestyle.

## Results and discussion

### The 4 *Pseudomonas* strains are able to form biofilms on wheat roots

**Fig. 1:**
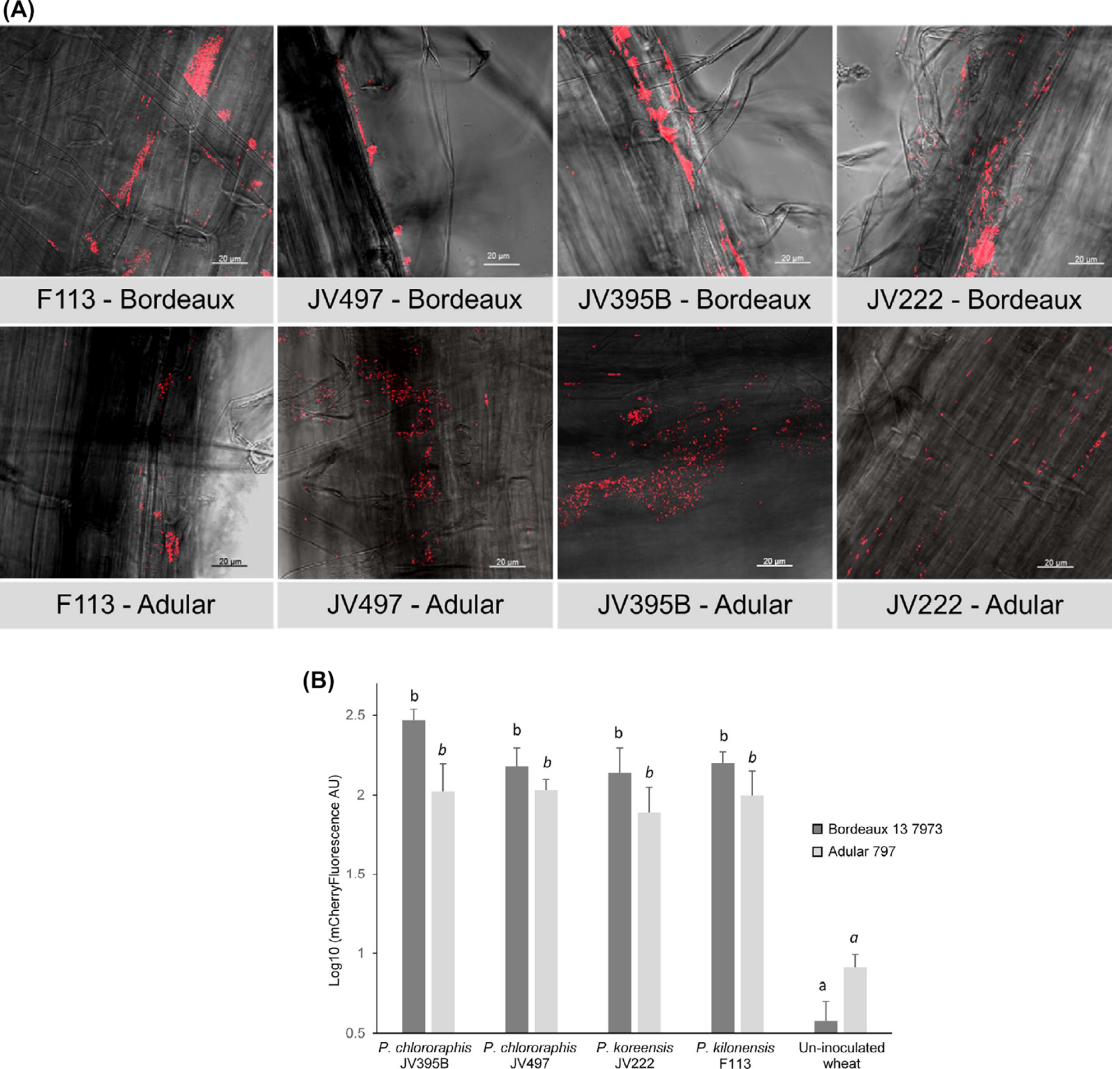
Confocal laser microscopy images (A) and semi‐quantitative analysis (B) of root colonization by *P. kilonensis* F113, *P. chlororaphis* JV497 and JV395B and *P. koreensis* JV222 on wheat genotypes Bordeaux 13 7973 and Adular 797, 7 days after bacterial inoculation. Photographs (A) were taken using a confocal laser scanning microscope with an excitation of 561 nm and an emission filter of 570–636 nm. Cells expressing mCherry are red and grey backgrounds correspond to root views observed with transmitted light. Images are representative of the analysis of 15 images per condition. Scale bars measure 20 µm. Root colonization (B) was estimated by quantifying the mCherry fluorescence recovered from roots, with an Infinite M200 PRO microplate reader using an excitation wavelength of 587 nm, and an emission wavelength of 661 nm. For all strains, significant higher levels of fluorescence were measured in the inoculated plants than in the un‐inoculated plants (Kruskal and Wallis test, *P*‐value < 0.05; different letters indicate significant differences).

First, the ability of the four studied strains to colonize and form biofilms on plant roots was studied. Root colonization of wheat was analysed 7 days after inoculation of each *Pseudomonas* strain on 2 wheat cultivars, Adular and Bordeaux. Confocal laser scanning microscopic observations and fluorescence colonization quantification showed that all *Pseudomonas* strains are able to colonize wheat roots (Fig. 1). The 4 *Pseudomonas* strains formed small or thick aggregates of cells throughout the root system and appeared as good wheat colonizers, as already reported for F113 (Valente *et al*., 2020).

### Numerous potential biosynthesis pathways of secondary metabolites were evidenced in the 4 root‐colonizing *Pseudomonas* strains by genome mining

To predict the putative biosynthesis pathways of *Pseudomonas* secondary metabolites shared by the 4 *Pseudomonas* strains, their genomes were screened using AntiSMASH and by implementing BLASTp search with ORFs involved in the biosynthesis of secondary metabolites produced by model strains of *Pseudomonas* (Table 1). Genome mining revealed that the 4 *Pseudomonas* strains harbour 20 gene clusters coding for the biosynthesis of bioactive secondary metabolites, some being involved in plant stimulation or biocontrol properties.

**Table 1:** Secondary metabolites and corresponding gene clusters of *Pseudomonas* strains.

Secondary metabolites	Gene involved in biosynthesis	Reference strain^a^	Locus number	JV222	JV395B	JV497	F113
Antimicrobial compounds							
2,4‐diacetylphloroglucinol (DAPG)	*phlIEDBCAFGH*	*P. kilonensis* F113	PSF113_2457‐2464				X
Hydrogen cyanide (HCN)	*hcnABC*	*P. kilonensis* F113	PSF113_2367‐2369				X
		*P. chlororaphis* subsp. aureofaciens 30‐84	JV395B_v1_740005‐740007		X		
			JV497_v1_90035‐90037			X	
		*Pseudomonas fluorescens* Pf0‐1	JV222_v1_460055‐4600557	X			
Pyrrolnitrin	*prnDCBA*	*P. chlororaphis* subsp. *aureofaciens* 30‐84	JV497_v1_130011‐130014			X	
			JV395B_v1_1150076‐1150079		*X*		
Phenazine	*phzIRABCABFD* + *phzH*	*P. chlororaphis* subsp. *aurantiaca* Pcho10	JV497_v1_250066‐250076			X	
	*phzIRABCABFD* + *phzO*	*P. chlororaphis* subsp. *aureofaciens* 30‐84	JV395B_v1_1530032‐1530043		X		
Alkylresorcinol	*darR; darCBA*	*P. chlororaphis* subsp. *aureofaciens* 30‐84	JV497_v1_210011‐210015			X	
			JV395B_v1_120011;v1_1280003‐1280005		X		
Putative Lankacidin	*lkcABCDEFGJ; pqqFABCDEG*	*P. kilonensis* F113	PSF113_3657‐3666; PSF113_5383‐5388				X
Quorum sensing related compounds							
Homoserine lactone	*phzI/phzR*	*P. chlororaphis* subsp. aureofaciens 30‐84	JV497_v1_250066‐250067			X	
			JV395B_v1_1530032‐1530033		X		
	*csaI/csaR*	*P. chlororaphis* subsp. aureofaciens 30‐84	JV497_v1_100065‐100066			X	
			JV395B_v1_760017‐760018		X		
	*aurI/aurR*	*P. chlororaphis* subsp. *aurantiaca* StFRB508	JV395B_v1_1510126‐1510127		X		
Auxin compounds							
Auxin	*iaaM*	*Pseudomonas moraviensis* R28‐S	JV222_v1_690002	X			
	*iaaM*	*P. chlororaphis* subsp. aureofaciens 30‐84	JV497_v1_320416			X	
			JV395B_v1_1150001		X		
	*iaaM; amiE*	*P. kilonensis* F113	PSF113_5381; PSF113_2053				X
Siderophore type compounds							
Pyridine‐2,6‐thiocarboxylic acid (PDTC)	*pdtCKPELMFGHIJON*	*P. kilonensis* F113	PSF113_2605‐2618				X
Pyoverdine	*pvdSL; pvdIJKDEONMP; fpvI; pvdA*	*P. kilonensis* F113	PSF113_1749‐1750; PSF113_1836‐1847; PSF113_1856‐1860				X
	*pvdSL; pvdLY; pvdAPMNOFEJID*	*P. chlororaphis* subsp. aureofaciens 30‐84	JV395B_v1_1300021‐1300023; JV395B_v1_1250001‐1270001		X		
			JV497_v1_201066‐201068; JV497_v1_200106‐210003			X	
	*pvdLS; pvdG‐pvdJ‐pvdO‐pvdP; pvdQfecR; fpvIpvdA*	*Pseudomonas moraviensis* R28‐S	JV222_v1_510035‐510036; JV222_v1_260099‐260112; JV222_v1_330053‐330053; JV222_v1_270006‐270007	X			
Achromobactin	*cbrDCBA; acsABC; yhcA; acsEDF*	*P. chlororaphis* subsp. aureofaciens 30‐84	JV395B_v1_940060‐JV395B_v1_940070		X		
			JV497_v1_120382‐120392			X	

Compounds	MIBiG	Locus number	JV222	JV395B	JV497	F113
Other antismash PKS and NRPS prediction (AntiSMASH)						
NRP1	–	JV395B_v1_910035		X		
		JV497_v1_120137			X	
NRP2	–	JV395B_v1_800032‐800033		X		
NRP3	–	JV497_v1_120298			X	
NRP4	BGC0001135	JV222_v1_330154‐330156	X			
NRP5	–	JV222_v1_260096‐260103	X			
NRP6	BGC0000438	JV222_v1_310040‐310042	X			
NRP7‐like	BGC0000387	JV222_v1_30009	X			
		JV395B_v1_10008		X		
		JV497_v1_10007			X	
		PSF113_0201				X
PK1	BGC0000837	JV395B_v1_90058‐90061		X		
		JV497_v1_30101‐30107			X	
		JV222_v1_100073‐100076	X			
		PSF113_0458‐0461				X
PK2	Butyrolactone	PSF113_3047‐3048				X

^a^ Annotation of BGCs for F113 was performed according to Redondo‐Nieto *et al. *(2013). For *P. chlororaphis* strains JV395B and JV497, annotation was made by comparison (coverage > 80%; identity > 80%) with protein sequences of the reference strains *P. chlororaphis* subsp. *aureofaciens* 30‐84 (Loper *et al.*, 2012), *P. chlororaphis* subsp. *aurantiaca* Pcho10 (Hu *et al.*, 2014) or StFRB508 (Morohoshi *et al.*, 2013). For *P. koreensis* JV222, *Pseudomonas moraviensis* R28‐S (Hunter *et al.*, 2014) or *Pseudomonas fluorescens* Pf0‐1 (Silby *et al.*, 2009) were used.

Indeed, all strains share biosynthetic gene clusters (BGCs) involved in the production of indolic compounds involved in root development like auxin derivatives, the volatile antimicrobial compound hydrogen cyanide (HCN) and pyoverdine (Table 1).

*P. kilonensis* F113 possesses *pdt* genes encoding the production of another siderophore, the pyridine‐2,6‐thiocarboxylic acid (PDTC, Table 1; Redondo‐Nieto *et al*., 2013). PDTC can chelate several metals other than iron (Zawadzka *et al*., 2006) and may protect F113 from the toxic effects of metals (Cortese *et al*., 2002), but its potential role in the rhizosphere fitness of F113 has never been established. F113 also harbours BGCs for the biosynthesis of the antimicrobial compounds lankacidin (*lkc* genes) and 2,4‐diacetylphloroglucinol (DAPG; *phl* genes). The *phl* operon is absent in the *P. choloraphis* and *P. koreensis* strains (Table 1). This result is consistent with a previous phylogenetic study (Almario *et al*., 2017). DAPG has a large spectrum of antimicrobial properties against bacteria, fungi and helminths (Couillerot *et al*., 2009). At low concentrations, it has signalling properties on host plant (Brazelton *et al*., 2008) or other bacteria (Combes‐Meynet *et al*., 2011).

The two *P. chlororaphis* strains, JV395B and JV497, share similar genetic clusters for the biosynthesis of pyrrolnitrin, phenazine and alkylresorcinol and of the siderophores, pyoverdine and achromobactin (Table 1). One difference between the two *P. chlororaphis* strains concerns the end of their phenazine BGCs where *phzH* for JV497 and *phzO* for JV395B are present. This difference may lead to the synthesis of different phenazine derivatives. *phzO* encodes a monooxygenase (Yu *et al*., 2018) leading to the production of hydroxylate derivatives of the PCA (2‐OH‐PCA, 2‐OH‐PHZ and putative di‐OH‐PCA; Maddula *et al*., 2008), whereas *phzH* encodes a transamidase transforming PCA into phenazine‐1‐carboxamide (PCN; Price‐Whelan *et al*., 2006). Phenazines represent a large class of natural antibiotics that exhibit broad‐spectrum antibiotic activity (Mavrodi *et al*., 2013). Their production is a primary mechanism involved into pathogen inhibition and contributes also to *P. chlororaphis* rhizosphere colonization and establishment (Mazzola *et al*., 1992). Unlike *P. kilonensis* and *P. koreensis,* the *P. chlororaphis* strains (JV395B and JV497) possess in their genomes the quorum sensing (QS) regulation genes *phzI/phzR* and *csaI/csaR*. JV395B owns a third QS regulation system *aurI/aurR* that is absent in JV497 genome (Table 1). These three QS systems take part in different regulation systems; *phzI/phzR* and *aurI/aurR* are implicated in phenazine biosynthesis regulation (Maddula *et al*., 2008; Morohoshi *et al*., 2017) whereas *csaI/csaR* controls the production of bacterial cell surface properties (Zhang and Pierson, 2001; Maddula *et al*., 2006).

AntiSMASH analysis allowed the detection of other non‐ribosomal peptide synthases (NRPS) and polyketide synthases (PKS) in every strain (Table 1), but in‐depth investigation on these BCGs does not allow to clearly identify the compounds synthesized. However, NRPS involved in the synthesis of putative syringopeptin (BGC0000438) and bicornutin (BGC0001135) were identified in strain JV222, putative butyrolactone in F113, mangotoxin (BGC0000387) and arylpropylene (BGC0000837) in all strains.

### Metabolomes of *Pseudomonas* strains differ according to biofilm and planktonic lifestyles

**Fig. 2:**
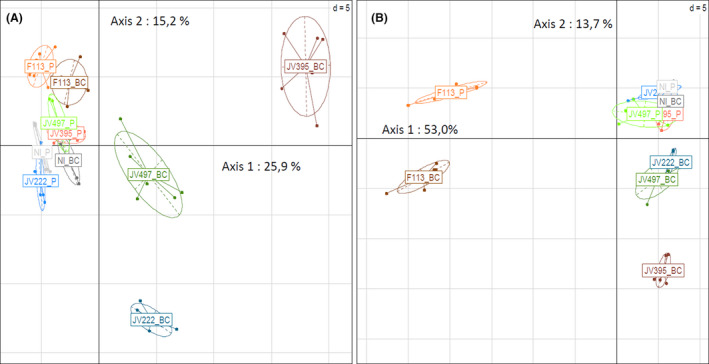
Principal component analysis of LC‐HRMS data obtained for the four *Pseudomonas* strains cultivated in planktonic (P) or in biofilm colony (BC) conditions after 6 days of incubation, based on intensity of ions (expressed in log10) in positive (A) and negative (B) modes. Relationships between metabolomes of the 4 fluorescent *Pseudomonas* strains, *P. chlororaphis* JV395B and JV497, *P. kilonensis* F113 and *P. koreensis* JV222, according to lifestyles are visualized along principal components PC1 = 25.9% and PC2 = 15.2% (*N* = 60 samples; 186 molecular ions) for the positive mode (A) and PC1 = 53.0% and PC2 = 13.7% (*N* = 60 samples; 230 molecular ions) for the negative mode (B).

After having evidenced the ability of the four plant‐associated *Pseudomonas* to form biofilms on wheat roots, a portion of metabolomes (i.e. compounds < 1000 Da) of the four *Pseudomonas* strains were compared between biofilm and planktonic cultures. Secondary metabolites constitute the major part of the metabolome of an organism and generally greatly exceed the number of primary metabolites (Moco *et al*., 2007). They are involved in the adaptation of organisms to their biotic and abiotic environment (Demain, 1999). The analytical setup developed in this study was focused on small metabolites and allowed detection of hundreds of compounds. Multivariate analysis was conducted on data obtained in negative and positive ionization modes (Fig. 2A and B); this allows the detection of different molecules regarding their atomic composition and chemical structure (Dettmer *et al*., 2007).

First, biological replicates from a same modality are clustered on the principal component analysis, revealing good repeatability. Second, in positive detection mode (Fig. 2A), axis 1, which represents 25.9% of the data variability, separated all strains according to their bacterial lifestyle, specifically for *P. chlororaphis* JV395B, *P. chlororaphis* JV497 and *P. koreensis* JV222. Axis 2, which represents 15.2% of the variability, separated strains from biofilm colony between each other. The *P. kilonensis* F113 strain was the only one that could be separated from the other strains in the planktonic lifestyle. Third, in the negative detection mode, *P. kilonensis* F113 (from biofilm and planktonic cultures) was separated from the other strains according to axis 1 (53%), suggesting that F113 can produce discriminant compounds, different from other strains (Fig. 2B). Axis 2, which represents 13.7% of the variability, separated each strain according to planktonic and biofilm colony lifestyle, as similarly observed for the positive ionization mode (Fig. 2A). Thus, in both ionization modes, strains were separated according to their lifestyle. Thus, metabolomes of the 4 studied *Pseudomonas* strains are strongly influenced by bacterial lifestyle. Moreover, strains separation was much more important in biofilm colony than in planktonic lifestyle especially for *P. koreensis* JV222, *P. chlororaphis* JV395B and *P. chlororaphis* JV497, indicating that these strains produce more specialized metabolites in biofilm. Several studies have previously shown that the physiology of bacterial cells differs between biofilm and planktonic lifestyles using transcriptomic (Waite *et al*., 2005), proteomic (Mikkelsen *et al*., 2007; Favre *et al*., 2018) or metabolomics approaches (Gjersing *et al*., 2007; Stipetic *et al*., 2016; Favre *et al*., 2018).

**Fig. 3:**
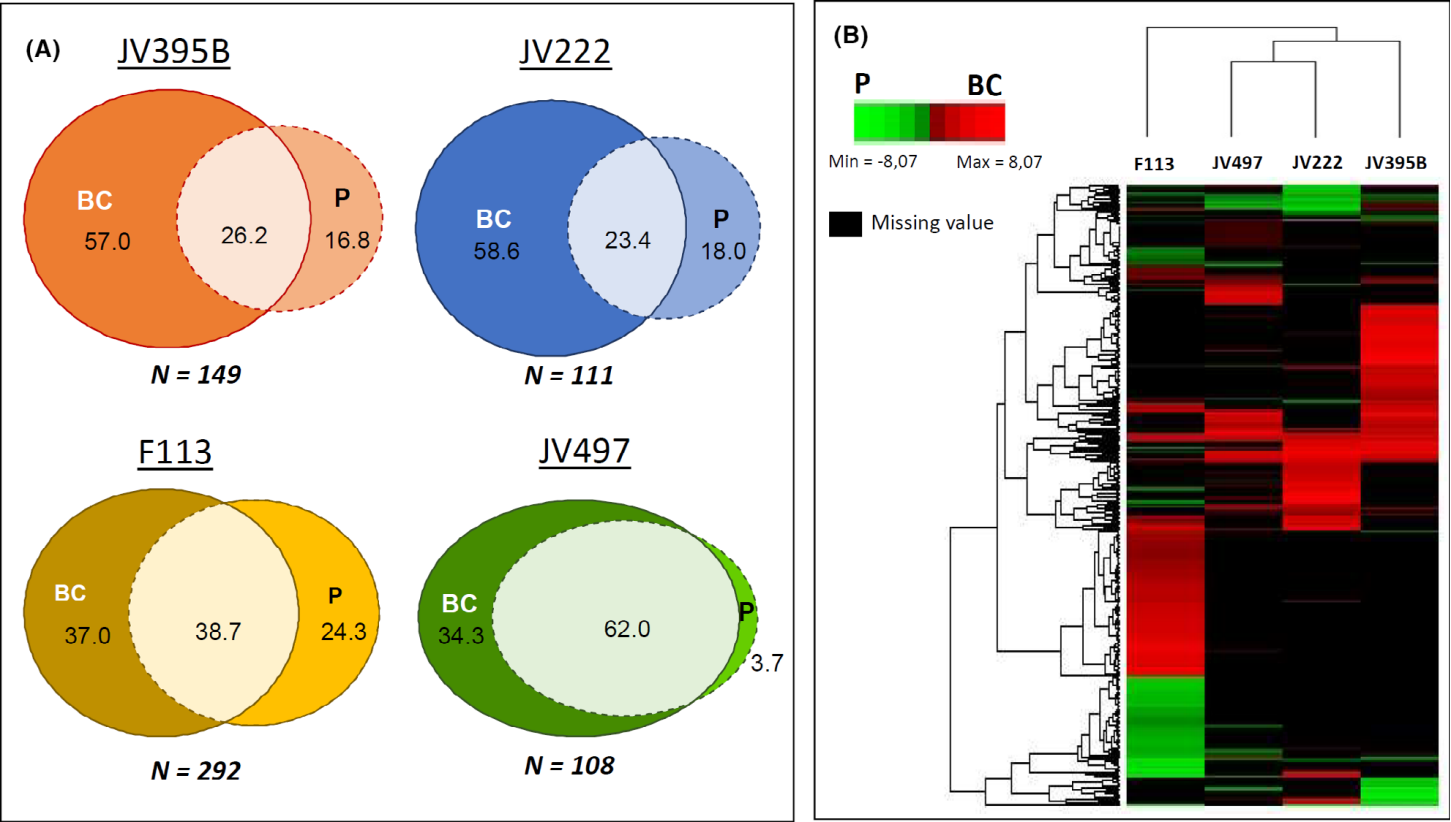
Venn diagrams representing the percentage of secondary metabolites significantly overproduced in biofilm colony (BC), planktonic (P) or shared between the two culture modes for each *Pseudomonas* strains, *P. chlororaphis* JV395B and JV497, *P. kilonensis* F113 and *P. koreensis* JV222 (*P*‐value < 0.05, Wilcoxon test) (A). Heatmap clustering of the secondary metabolites differentially produced in biofilm colony (red) or in planktonic (green) cultures (*P*‐value < 0.05, Wilcoxon test) (B). The map was generated using log2 fold change values calculated from the metabolome table matrix constituted of 328 *m/z* features.

To compare bacterial secondary metabolites differentially produced by each strain according to the bacterial lifestyle conditions, univariates analyses were performed (Fig. 3). Ions detected in positive and negative ionization modes were combined in order to have one ion per metabolite. Different behaviours were highlighted for every strain. For *P. kilonensis* F113, *P. koreensis* JV222 and *P. chlororaphis* JV395B, most of metabolites were significantly differentially produced between planktonic and biofilm colony lifestyle (61.3% of them for F113, 73.8% for JV395B and 76.6% for JV222; Fig. 3A). *P. chlororaphis* JV395B and *P. koreensis* JV222 metabolomes were the most influenced by bacterial lifestyle (Fig. 3A) with more than half of their metabolites overproduced in biofilm. In *P. chlororaphis* JV497, only 38% of them were differentially produced between the tested conditions (Fig. 3A). But it is important to note that some of the most abundant compounds produced by *P. chlororaphis* JV497 were more produced in biofilm condition.

Thus, for every strain, most of the detected compounds presented higher intensity in biofilm than in planktonic culture (Fig. 3A and B). *P. koreensis* JV222, *P. chlororaphis* JV395B and *P. chlororaphis* JV497 produced much more metabolites with a significant higher amount in biofilm colony than in planktonic cultures (i.e. 3.3; 3.4 and 9.2 times more metabolites in biofilm, respectively; Fig. 3A and B). For *P. kilonensis* F113, the difference was lower with only 1.5 times more metabolites overproduced in biofilm colony. The chemical diversity of the secondary metabolites differently produced according to bacterial lifestyle is shown for every strain on a heat map (Fig. 3B). *P. chlororaphis* strains JV395B and JV497, and *P. koreensis* JV222 are clustered together, whereas *P. kilonensis* strain F113 is outside this cluster. Indeed, F113 showed discriminant metabolites from the other strains in both planktonic culture (green) and biofilm colony (red; Fig. 3B).

### Most *Pseudomonas* bioactive secondary metabolites, whose BCGs were predicted by genome mining and molecular networking, are overproduced in biofilms

**Fig. 4:**
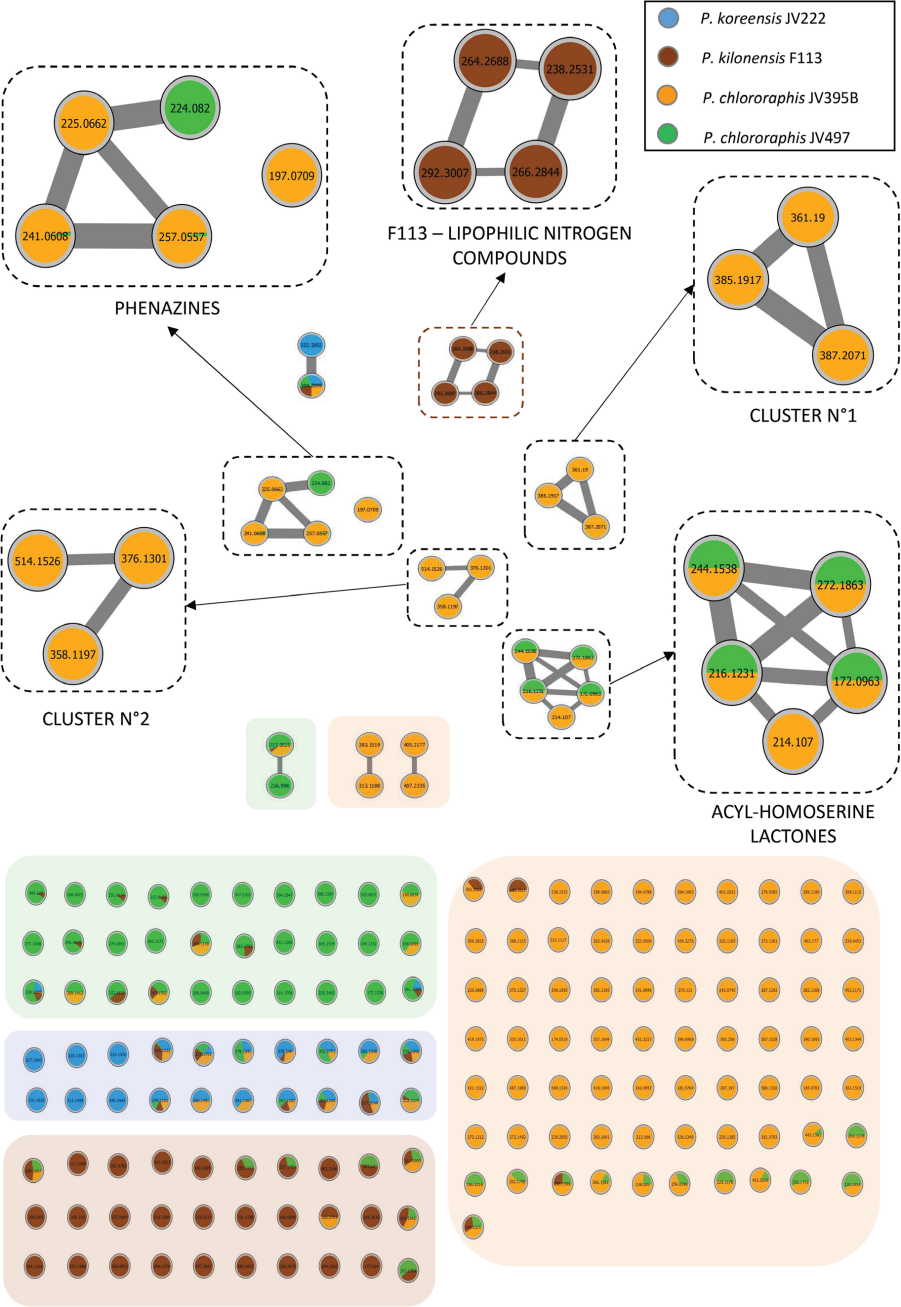
Molecular networks of MS/MS data obtained from biofilm cultures of the four bacterial strains *P. chlororaphis* JV395B (orange), *P. chlororaphis* JV497 (green), *P. kilonensis* F113 (brown) and *P. koreensis* JV222 (blue), using a cosine similarity cut‐off of 0.6. Cluster position was determined according to t‐distributed stochastic neighbour embedding (t‐SNE) output. Nodes are labelled with parent *m/z* ratio. Relative proportion of the compounds belonging to each strain was represented as a pie chart in each node. This figure highlights that most single ions from the 4 *Pseudomonas* strains in biofilms (i.e*. P. chlororaphis* JV395B (orange box), *P. chlororaphis* JV497 (green box), *P. kilonensis* F113 (brown box) and *P. koreensis* JV222 (blue box)) are unique to each strain. Clusters constituted of more than 3 ions are emphasized.

Bioactive secondary metabolites, whose BCGs were predicted by genome mining, were sought in planktonic and biofilm cultures (Tables 1 and 2). Only thirty‐eight metabolites could be annotated in the *Pseudomonas* biofilm and planktonic cultures (Table 2). While biosynthetic gene clusters involved in the production of different metabolites were predicted in genomes of the *Pseudomonas* strains (i.e. achromobactin, lankacidin, alkylresorcinol, HCN and pyoverdine), these compounds were not produced under the tested conditions, or were not detectable with our analytical method. To go further, a molecular networking (MN) approach was done on biofilm‐produced secondary metabolites in order to cluster metabolites that share same MS/MS fragmentation (Watrous *et al*., 2012). MN evidenced 5 clusters that contain more than two metabolites (Fig. 4). The majority of biofilm‐produced metabolites clustered on the MN are synthesized by the *P. chlororaphis* strains and belong to AHL and phenazine families (Fig. 4 and Table 2). Two other clusters (N°1 and N°2) consisting of 3 nodes correspond to unknown compounds produced by JV395B and contain the ions *m/z* 514.1526, 376.1297 and 358.1197 (cluster n°1), and 385.1917, 387.2076 and 361.1900 (cluster n°2). The last cluster (4 nodes) corresponds to lipophilic nitrogen compounds produced by F113 (Fig. 4 and Table 2). Four other clusters are constituted of only 2 metabolites that could not be assigned to a chemical family (Fig. 4). Finally, most of the ions not grouped in clusters were produced by only one strain confirming that the 4 strains had different metabolomes in biofilms (Figs 2 and 4).

**Table 2:**
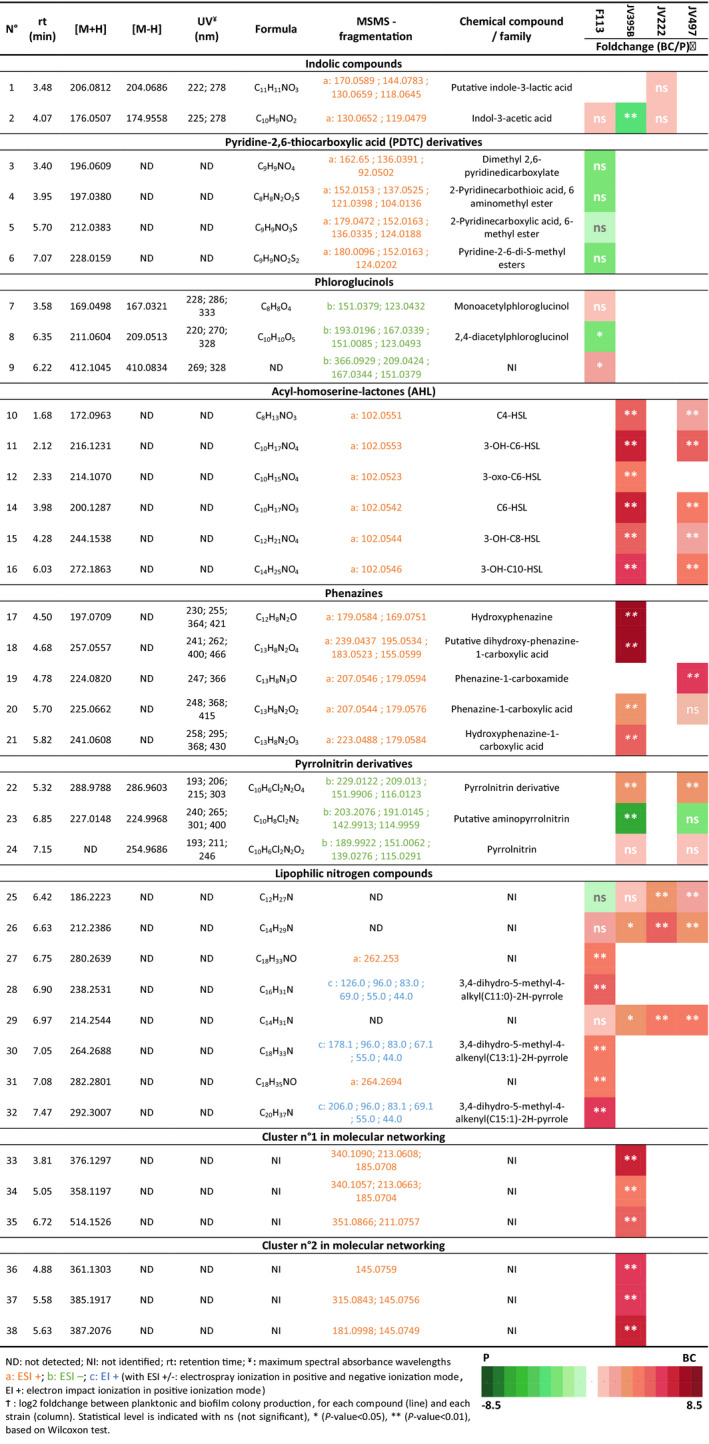
Chemical characterization of annotated secondary metabolites combined with heatmap representation of their differential production in planktonic or biofilm colony cultures.

First, several metabolites were observed to be distinctly produced by the *Pseudomonas* strains but showed little difference between biofilm/planktonic lifestyle, or were even repressed in biofilm conditions. DAPG, the monoacetylphloroglucinol (MAPG) and another putative compound that shared closed UV spectra and MS/MS fragmentation as DAPG were only detected in F113 culture (Table 2). These compounds were weakly or not influenced by bacterial lifestyle in *P. kilonensis* F113. DAPG production in fluorescent *Pseudomonas* strains is known to be regulated by other biotic factors like the presence of the bacterial predator *Acanthamoeba castellani* (Jousset and Bonkowski, 2010) or plant compounds (de Werra *et al*., 2008).

Indole‐3‐acetic acid (IAA) produced through *iaaM* in the studied *Pseudomonas* strains was detected in *P. kilonensis* F113, *P. koreensis* JV222 and *P. chlororaphis* JV395B cultures but not in *P. chlororaphis* JV497 ones. IAA is the most abundant naturally occurring auxin, which is a class of phytohormones involved in the regulation of plant growth and development (Szkop and Bielawski, 2013). IAA was produced in similar proportions independently of the bacterial lifestyle in F113 and JV222, whereas it was significantly more produced in planktonic culture than biofilm colony in JV395B. *P. koreensis* JV222 also produced indole‐3‐lactic acid in same proportions, in biofilm and planktonic cultures. IAA has been shown to stimulate the formation of bacterial biofilm at low concentrations, whereas it has negative effect on biofilm formation at high concentrations (Plyuta *et al*., 2013). Another indolic compound, the intracellular signal indole, also presents a discrepancy of effects on the control of biofilm formation depending on bacterial strains and concentration (Lee and Lee, 2010). Thus, it is not surprising that IAA production is not similarly controlled according to bacterial lifestyle in the different *Pseudomonas* tested strains.

This analysis also led to the detection of thioester and acid derivatives of PDTC in *P. kilonensis* F113 cultures (Table 2), but these compounds were not significantly differentially produced in biofilm and planktonic cultures. Its biosynthetic gene cluster was predicted in F113 genome by Redondo‐Nieto *et al*. in 2013 but the metabolite was never detected in F113 cultures. Compounds highlighted here were ester, thioester and acid derivatives of PDTC but not PDTC itself (Table 2; Fig. S2; Budzikiewicz *et al*., 1981). PDTC is not stable in solution (Cortese *et al*., 2002), but the detection of PDTC derivatives in F113 cultures can suggest the potential ability of F113 to produce PDTC (Budzikiewicz, 2003).

The production of pyrrolnitrin, a compound with a strong antifungal activity *in vitro* (Selin *et al*., 2010) known to be produced by some fluorescent *Pseudomonas* species like *P. chlorororaphis* or *P. protegens,* was not significantly changed in *P. chlororaphis* JV395B and JV497 according to the bacterial mode of life (Table 2). However, pyrrolnitrin derivatives are influenced by bacterial lifestyle. Putative aminopyrrolnitrin (i.e. pyrrolnitrin immediate precursor in the biosynthesis pathway) was significantly more produced by *P. chlororaphis* JV395B in planktonic mode, while another pyrrolnitrin derivative (compound n° 22 in Table 2) was significantly more produced in biofilm in both *P. chlororaphis* strains.

Second, among the thirty‐eight annotated compounds twenty‐five were observed clearly overproduced in biofilm cells. Five AHLs derivatives (i.e. C_4_‐homoserine lactone (HSL), 3‐OH‐C_6_‐HSL, C_6_‐HSL, 3‐OH‐C_8_‐HSL and 3‐OH‐C_10_‐HSL) were produced by both *P. chlororaphis* strains; only 3‐oxo‐C_6_‐HSL was not produced by JV497 (Fig. 4), probably because JV497 does not possess the *aurI/aurR* regulation system that was strongly implicated in 3‐oxo‐C_6_‐HSL production (Morohoshi *et al*., 2017). AHLs are signalling molecules released by bacteria in response to population size that induce the coordinated expression of specific genes when sensed by neighbouring cells (Keller and Surette, 2006; Flemming *et al*., 2016). All AHL derivatives were overproduced in biofilm colony (Table 2), as commonly described in literature (Waite *et al*., 2005). 3‐OH‐C_6_‐HSL was for instance 124 and 24 times more produced in biofilm colony than in planktonic cultures in *P. chlororaphis* JV395B and *P. chlororaphis* JV497 respectively. By providing a closed system in which signalling molecules can be concentrated, the biofilm is an environment that facilitates intercellular signalling, which may explain the observed higher AHL concentrations in *P. chlororaphis* biofilm cultures (Charlton *et al*., 2000; Flemming *et al*., 2016). AHLs overproduction in biofilm may modify the physiology of bacterial cells, the biocontrol secondary metabolites they produce (particularly phenazines and pyrrolnitrin) and as a consequence their biotic interactions within the rhizosphere community (Venturi and Keel, 2016). This enhanced production may also directly impact the host plant, since AHL derivatives can directly interact with plant receptors and subsequently modify gene expression in plants (Schikora *et al*., 2016; Besset‐Manzoni *et al*., 2018). Nevertheless, AHLs were probably not the only regulation system. Indeed, although JV222 and F113 shared a distinct metabolism according to bacterial lifestyle, no AHL derivatives was detected in their cultures. However, Laue *et al*. in 2000 described the presence of 3 AHLs (i.e. C_6_‐HSL, C_10_‐HSL and 3OH‐C_14:1_‐HSL) in *P. kilonensis* F113 supernatant fractions produced by HdtS, a putative novel *N*‐acyl‐homoserine lactone synthase, which does not belong to the LuxI or LuxM family of AHL synthases. Even though our protocol allowed the detection of AHLs in *P. chlororaphis* strains JV395B and JV497, none of the above‐cited AHLs were detected in *P. kilonensis* F113 cultures under the tested conditions. In 2005, HdtS was rather described as a lysophosphatidate acyltransferase (LPA) and not as an AHL synthase (Cullinane *et al*., 2005). It is conceivable that other cell density‐dependent regulation systems than AHLs might be implicated in secondary metabolism regulation in F113 or JV222 biofilms.

Other compounds strongly influenced by bacterial lifestyle were phenazines. *P. chlororaphis* JV395B and *P. chlororaphis* JV497 did not produce the same phenazine derivatives. Indeed, both strains produced phenazine‐1‐carboxylic acid (PCA), but *P. chlororaphis* JV395B produced hydroxyl derivatives of PCA, like 2‐hydroxyphenazine‐1‐carboxylic acid (OH‐PCA), putative dihydroxy‐phenazine‐1‐carboxylic acid (di‐OH‐PCA) and 2‐hydroxyphenazine (OH‐Phz), whereas *P. chlororaphis* JV497 produced an aminated derivative, phenazine‐1‐carboxamide (PCN; Fig. 4). In our analysis, their intensities were closed to background noise in planktonic cultures, while they became major compounds in biofilm colony, with different proportions according to chemical derivatives (Table 2). Indeed, *P. chlororaphis* JV395B produced 6 times more PCA, 18.3 times more OH‐PCA and 229.1 times more OH‐Phz in biofilm colony than in the planktonic lifestyle. In the case of JV497, it produced low amount of PCA; most of this compound was transformed into PCN that is 56 times more produced in biofilm colony. Phenazines have antimicrobial activity and play a complex role in the ecology and lifestyle of organisms, including in biofilms (Price‐Whelan *et al*., 2006; Selin *et al*., 2010). Their biological activity depends on the chemical structure of phenazine derivatives. Maddula and collaborators in 2008 showed that more efficient conversion of PCA into OH‐PCA resulted in better initial biofilm attachment and significant changes in mature biofilm architecture, OH‐PCA being more produced in biofilms than PCA. Both phenazines and AHLs corresponded to main discriminant compounds separating *P. chlororaphis* biofilm and planktonic cells on the principal component analysis (Fig. 2). Phenazine production is under the control of quorum sensing regulation systems *phzI/phzR* and *aurI/aurR* (Maddula *et al*., 2006), especially by 3‐OH‐C_6_‐HSL (Morohoshi *et al*., 2017). It can therefore be assumed that enhanced quorum sensing signalling in biofilm colony was related to increased phenazine production in biofilm.

Finally, 8 lipophilic nitrogen metabolites belonging to the same MN cluster more produced in biofilm were detected in *Pseudomonas* cultures (i.e. 25.6‐, 13.7‐ and 44.6‐fold for compounds 28, 30 and 32; Fig. 4). Three of these compounds were characterized as 3,4‐dihydro‐5‐methyl‐4‐alkyl(C11:0)‐pyrrole, 3,4‐dihydro‐5‐methyl‐4‐alkenyl(C13:1)‐2H‐pyrrole and 3,4‐dihydro‐5‐methyl‐4‐alkenyl(C15:1)‐2H‐pyrrole (compounds n° 28, 30 and 32 in Table 2; Fig. 4; Fig. S2). There is no information on the biological activity of these derivatives of 1‐pyrroline, but we know they have been previously isolated in *P. putida* DSM 3601 cultures (Hildebrand and Budzikiewicz, 1986). Compounds 25, 26 and 29 were produced by the 4 *Pseudomonas* strains whereas compounds 27, 28 and 30 to 32 were only produced by F113. Six other metabolites produced by *P. chlororaphis* JV395B corresponding to MN clusters n°1 and n°2 (Fig. 4) were also strongly overproduced in biofilm but we were unable to chemically identify them.

## Concluding remarks

**Fig. 5:**
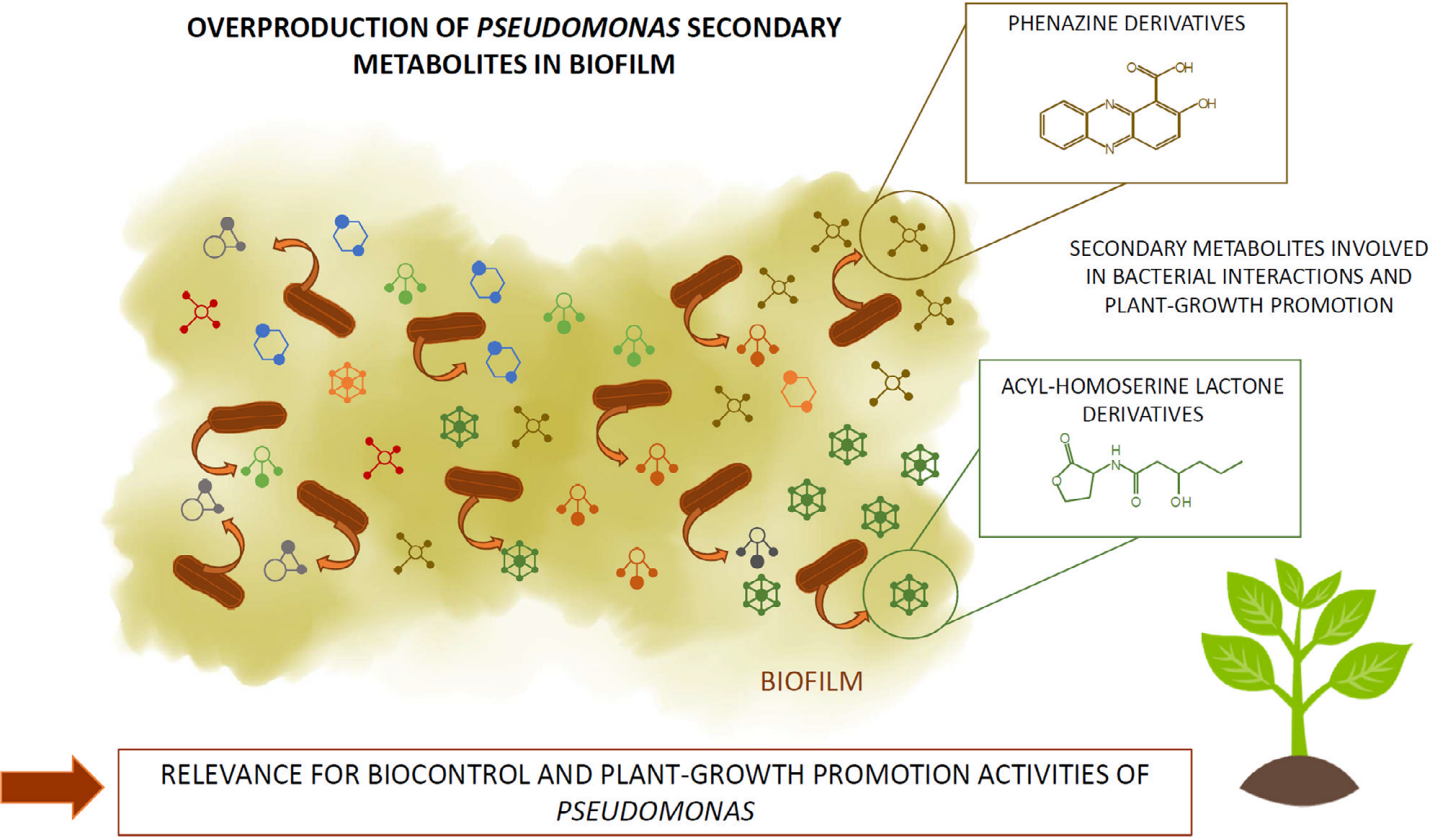
*Pseudomonas* strains produce a higher diversity of secondary metabolites in biofilm colonies than in planktonic lifestyle. In particular, the biosynthesis of key compounds implicated in bacterial biotic interactions like AHLs or phenazines is strongly enhanced in biofilms. Biofilms are thus key ways to enhance the biocontrol and biostimulant activities of bioinoculant for agriculture purposes.

Plant‐associated bacteria can colonize roots under a wide range of conditions by forming biofilms. Biofilm cells are clearly physiologically distinct from free‐living bacterial cells and express emergent properties that are not predictable from the study of free‐living bacterial cells (Stoodley *et al*., 2002). In the present study, we combined genome mining and comparative metabolomics analyses to characterize the metabolomes of four plant‐associated *Pseudomonas* strains in biofilm and planktonic conditions. We have highlighted that a higher diversity of *Pseudomonas* secondary metabolites were produced in biofilm colony than in planktonic lifestyle. The majority of metabolites were still uncharacterized but some of the major compounds implicated in bacterial biotic interactions like AHLs or phenazines were strongly enhanced in biofilms. It is reasonable to think that the enhanced biosynthesis of secondary metabolites in biofilm will influence the biotic interactions of fluorescent *Pseudomonas* with their host plant and with other microorganisms within the rhizosphere (Fig. 5). This work provides new relevant avenues regarding inoculum development in that biofilms should be considered as a critical way to enhance the biocontrol and biostimulant activity of bacteria, since biofilms lead to the enhanced biosynthesis of a broad diversity of secondary metabolites and of crucial elements for plant‐growth promotion.

## Experimental procedures

### Biological material and culture growth conditions

This study was performed on 4 strains belonging to the fluorescent *Pseudomonas* group: *Pseudomonas kilonensis* F113 isolated in 1992 from the sugar beet rhizosphere (Shanahan *et al*., 1992) and three *Pseudomonas* strains isolated in our laboratory in 2013 from bulk soil in maize planted fields, *Pseudomonas chlororaphis* JV395B and JV497 and *Pseudomonas koreensis* JV222 (Vacheron *et al*., 2016). *Pseudomonas* strains were routinely grown in King’s B (KB) agar medium (King *et al*., 1954) and Luria Bertani (LB) broth medium (Bertani, 1951) at 28°C. Gentamicin (Sigma Chemical, St. Louis, MO, USA) was used at 25 µg ml^−1^. Minimum medium (MM) was used for both planktonic and biofilm colony conditions, which is composed of fructose (14.4 g l^−1^), NH_4_Cl (1 g l^−1^), KH_2_PO_4_ (1.36 g l^−1^)/K_2_HPO_4_ (1.74 g l^−1^), MgSO_4_ (0.8 g l^−1^) and a mix of 15 amino acids (alanine, arginine, asparagine, aspartic acid, cysteine, glutamic acid, glycine, histidine, isoleucine, leucine, phenylalanine, threonine, tryptophan, tyrosine, valine at 0.01 mg ml^−1^ each). Fructose was used as carbon source because it is one of the dominant sugars in wheat root exudates (Jalali and Suryanarayana, 1971; Vančura and Hanzlíklová, 1972; Derrien *et al*., 2004), it is known to stimulate the biosynthesis of antimicrobial compounds in *Pseudomonas* (Shanahan *et al*., 1992; Duffy and Défago, 1999), and it allowed comparable growth of the four *Pseudomonas* strains (data not shown). Broth and agar MM medium only differ by the addition of agar 12 g l^−1^ (European biological agar, Pronadisa, Conda, Madrid, Spain).

### Analysis of bacterial root colonization by confocal laser scanning microscopy and fluorescence quantification

The production of biofilms by *Pseudomonas* strains on plant roots was performed on wheat cultivar Bordeaux 13 7973 and Adular 797 (INRA Clermont‐Ferrand GEDEC, France). All strains were tagged with mCherry*,* to monitor bacterial cell colonization by fluorescence microscopy. The plasmid pOT1eM from Meyer *et al*. (2018), obtained by cloning the *mCherry* gene under P_tac_ promoter control (constitutive expression), was introduced by electroporation, and tagged strains were selected on KB Gm25 at 28°C. Root colonization patterns of *Pseudomonas* strains were performed on wheat under gnotobiotic systems. Wheat seeds were surface sterilized as described by Pothier *et al*. (2007). Disinfected seeds were germinated on sterile plant agar medium (8 g l^−1^; Sigma Chemical) for 3 days in the dark at 28°C. Bacterial inoculants were grown overnight in liquid LB Gm25, at 28°C, and at 180 g. The cells were collected by centrifugation at 5000 g during 10 min, gently washed, suspended in MgSO_4_ 10 mM and the suspensions adjusted to the final required concentrations. Inoculation of each strain on wheat cultivar was done by adding 50 µl of a 10^7^ cells/mL suspension on germinated seeds (i.e. 5. 10^5^ cells/plant). The same volume of MgSO_4_ solution was added for uninoculated controls. Square plates (120 × 120 × 17 mm; Greiner Bio‐One, Stonehouse, UK) containing water agar plant (8 g l^−1^) and four seedlings were used. The plates were placed for 7 days in a growth chamber at 21°C with 16 h of light (150 lE/m^2^/s) and 8 h of dark, and hygrometry rate of 60%. For confocal laser scanning microscopy (CLSM), samples 1–2 cm in length were cut from apical root and hair root zones and mounted in Aqua‐Poly/Mount (Polysciences, Eppelheim, Germany). A microscope Confocal Zeiss LSM 800 (Carl Zeiss, Le Pecq, France) equipped with argon–krypton and He‐Ne lasers was used for analysis of red fluorescence emitted by bacteria (excitation at 587 nm and detection at 661 nm). Image treatment was performed using LSM software release 3.5 (Carl Zeiss). Five root systems were analysed per treatment, and 3 images per root system were taken. One representative view for each strain was presented in Figure 1A. In addition, root colonization by the *Pseudomonas* strains was estimated by quantifying the mCherry fluorescence recovered from roots as previously reported (Valente *et al*., 2020). Briefly, the roots were ground for 1 min with a FastPrep‐24 Classic Instrument (MP Biomedicals, Santa Ana, CA). The biggest plant debris was pelleted by centrifugation, and 200 μl of each supernatant were transferred in black 96‐well plates. The mCherry fluorescence intensity of supernatants was measured with an Infinite M200 PRO microplate reader (Tecan, Mannedorf, Switzerland), using an excitation wavelength of 587 nm and an emission wavelength of 661 nm. Non‐inoculated plantlets were used as controls (Fig. 1B).

### Genome analysis of *Pseudomonas* secondary metabolites

Genomic DNA extraction was done from an overnight culture of strains JV395B, JV497 and JV222, using a Nucleospin tissue kit (Macherey Nagel – 740952.50, Hoerdt, France). Genomic DNA was sequenced at Mr. DNA (Shallowater, TX, USA). Genome sequencing was performed using MiSeq Illumina technology generating a 2 × 300 bp paired‐end library. The NGen V14 (DNAStar) was used to trim sequences (default settings) and for *de novo* assembling (average size of *P. fluorescens* genomes as assembly parameters). Gene cluster coding for secondary metabolites was identified using the MicroScope web platform by BLAST comparison (Vallenet *et al*., 2017) with characterized secondary metabolite gene clusters and reference strains available in literature and using antiSMASH (Medema *et al*., 2011; Blin *et al*., 2017). Only clusters with reliable prediction were reported. Bacteriocins were not reported. Draft genomes of *Pseudomonas* strains have been deposited at the NCBI under BioProject ID PRJNA565121, sample SAMN12734735 for *Pseudomonas chlororaphis* JV395B, under BioProject ID PRJNA565122, sample SAMN12734736 for *Pseudomonas chlororaphis* JV497 and under BioProject PRJNA564967, sample SAMN12727389 for *Pseudomonas koreensis* JV222. Genome sequences of the de novo assembly have also been deposited at DDBJ/EMBL/GenBank under the accession numbers VWPB00000000 for strain JV395B, VWPC00000000 for strain JV497, and VWPA00000000 for strain J222. The accession number of *P. kilonensis* F113 genome is CP003150.1.

### Planktonic and biofilm colony culture conditions and metabolite extraction

Bacterial cultures were made either in liquid fructose minimal medium (MM; i.e. planktonic culture) or on MM agar (i.e. biofilm colony culture). Bacterial inoculants were obtained after overnight growth in LB medium and washed twice with MM broth. Experimentation was carried out in 24‐well plate (Cellstar^®^, Greiner Bio‐One, France). Distinct wells were used for biofilm cultures on MM agar and for planktonic cultures in MM broth. One millilitre of MM broth and of MM agar per well were inoculated with each of the four strains with an initial population of 5.10^6^ CFU ml^−1^. Then, plates were sealed with (AeraSeal^®^‐Dominique Dutscher, France) and incubated at 28°C, during 6 days. Biofilm colonies were formed at the air–agar interface (Cabeen *et al*., 2016). Uninoculated agar and broth media were also integrated as control conditions. All conditions were performed in 6 replicates leading to a total of 60 samples. Metabolite extractions were conducted on the whole bacterial cultures, that is bacterial cells and MM broth for planktonic cultures and bacterial biofilm colonies and MM agar for biofilm culture. Indeed, whole planktonic and biofilm cultures were transferred into Eppendorf© tubes and extracted with 750 µl of ethyl acetate. Liquid/liquid partition was implemented for broth medium, and solid/liquid extraction was done on divided pieces of agar (5 mm × 5 mm × 3 mm). Solid/liquid extraction was made by agitation at 150 g during 10 min and sonication 10 min, centrifugation and recovery of the supernatant. Liquid/liquid partition was performed by 10 min of agitation and recovery of the upper organic phase after a resting period of 5 min. The extraction protocol was repeated, giving a total extract volume of 1500 µl by sample. Then, the organic phase (ethyl acetate) was dried using a SpeedVac (Centrivap Cold Trap Concentrator LABCONCO). Dried extracts were suspended in 50 µl of methanol and centrifuged for 5 min at 12 000 g for both liquid and solid culture samples. The efficiency of the extraction method on planktonic and biofilm colony sample types was compared, thanks to the quantification of an internal standard, tryptophan, in un‐inoculated MM broth and MM agar. No difference was observed between the tryptophan concentrations recovered from broth and agar (*P‐*value = 0.21, Wilcoxon test, Fig. S1). Forty‐five microlitres were then transferred in vials for ultra‐high pressure liquid chromatography coupled with mass spectrometry (UHPLC‐MS) analysis. A quality control (QC) sample was prepared by mixing 2 µL of each sample previously described (60 samples) in order to control analytical repeatability during UHPLC‐MS analysis.

### Liquid chromatography coupled with high‐resolution mass spectrometry (LC‐HRMS) analysis

Secondary metabolites were analysed using Agilent Technologies^®^ Accurate‐Mass Q‐TOF LCMS 6530, with LC 1290 Infinity system. The separation was carried out at 40°C using a 120 EC‐C18 column (3.0 × 100 mm × 2.7 µm; Agilent Poroshell). Each sample (3 µl) was injected at the head of the column, and the column was eluted at 0.7 ml min^−1^ with a solvent gradient using solvent A (water with formic acid 0.4% (v/v)) and solvent B (acetonitrile). Proportion of solvent B increases, step by step, from 10% to 36% during 4.5 min; 36% to 100% during 4 min, followed by 2 min isocratic phase with 100% solvent B; back from 100% to 10% in 0.5 min and equilibration at 10% solvent B during 3 min until the end of the run at 14 min. Mass analyses were made in positive and negative mode, with the nebulization gas (Nitrogen) at a flow of 10 l min^−1^ and 40 psg pressure. The capillary tension was 3000 V and gave ionization energy of 100 eV. Analyses included QC and blank samples every 10 sample runs, giving a total of 8 blanks and 8 QCs. Moreover, in order to facilitate the identification of specific compounds and allow molecular network output, some samples were analysed with tandem mass spectrometry (MS/MS). MS/MS analyses were performed by collision‐induced dissociation (CID) with a collision energy of 20 eV. Chromatograms were explored with MassHunter Qualitative Analysis B.07.00 software (Agilent Technologies).

### Gas chromatography coupled with mass spectrometry (GC‐MS) analysis

Complementary GC‐MS analyses were conducted on each sample to allow metabolite identification, using the method described by Rozier *et al*. (2016). Aliquots of 1 μl were injected in split‐less mode using an Agilent 7000A EI triple Quad coupled with the 7890A GC system on Agilent 100915 – 433 column (HP5‐MS, 0.25 mm × 30 m × 0.25 µM). Chromatograms were explored with MassHunter Qualitative Analysis B.07.00 software.

### Data processing, statistical analysis, metabolite identification and molecular networking

Our metabolomic analysis workflow was focused on small molecules (< 1000 Da). LCMS raw files were converted to mzXML format using MS convert ‘ProteoWizard 2.1’ with filtering *m/z* outside the range of [50–450] seconds (Kessner *et al*., 2008). The data were then processed using both R software and the collaborative Galaxy platform ‘Workflow4metabolomics’ version 3.3 (Giacomoni *et al*., 2015). The overall workflow of data processing is as follows: (i) peak extraction (xcms R package 3.6.1; Smith *et al*., 2006), (ii) peak alignment (xcms R package 3.6.1), (iii) elimination of ions also found in blank samples, (iv) elimination of ions having a coefficient of variation (CV) of signal intensity higher than 25% in QC pool samples, (v) elimination of the isotopic ions or adducts that represent the same molecule (CAMERA R‐package and manual control validation; Kuhl *et al*., 2012), (vi) transformation of ions intensity into log10 and (vii) substitution of every log10 intensity value under 3.5 by a threshold of 3.5 in order to avoid interference due to missing value (feature not detected in a single class but detected in other classes; Di Guida *et al*., 2016). Then, after normalization and filtration steps, table matrix containing the intensity areas for each feature defined by the couple retention time and *m/z* was used for statistical analysis. All XCMS parameters were described in Table S1. Statistical analyses were realized using R logiciel 3.5.1©2018 software version. Principal component analyses were made with R package Ade4 (Thioulouse *et al*., 1997; Chessel *et al*., 2004). Non‐parametric univariate statistical analyses were conducted using Wilcoxon rank sum statistical test (*P*‐value = 0.05) on ion intensities for each strain in planktonic or biofilm colony lifestyle. Heatmap representation was done with the PermutMatrix software (Caraux and Pinloche, 2005). Identification of monoacetylphloroglucinol (MAPG), 2,4‐diacetylphloroglucinol (DAPG), pyrrolnitrin, indole‐3‐acetic acid (IAA), phenazine‐1‐carboxylic acid (PCA), 3‐OH‐C_6_‐HSL and dimethyl 2,6‐pyridinedicarboxylate were confirmed by comparison of the retention time and accurate mass of compounds in bacterial culture extracts with those of pure chemical standards (Table S1). Other phenazine, HSL or dimethyl 2,6‐pyridinedicarboxylate derivatives were characterized by implementing a molecular networking approach and by comparing their UV spectra, accurate mass and MS/MS fragmentations to those of standards of PCA, 3‐OH‐C_6_‐HSL and dimethyl 2,6‐pyridinedicarboxylate. Finally, 3,4‐dihydro‐5‐methyl‐4‐alkyl(C11:0)‐pyrrole, 3,4‐dihydro‐5‐methyl‐4‐alkenyl(C13:1)‐2H‐pyrrole and 3,4‐dihydro‐5‐methyl‐4‐alkenyl(C15:1)‐2H‐pyrrole were characterized by comparing mass spectrometry (MS) accurate mass and MS/MS fragmentation to spectral data from the publication of Hildebrand and Budzikiewicz (1986). Finally, in view to characterize *Pseudomonas* secondary metabolites produced in biofilm, a molecular networking (MN) approach was developed, thanks to Metgem software with cosine score = 0.6 (Olivon *et al*., 2018). MN was conducted on MS/MS data from *Pseudomonas* biofilm extracts according to the protocol described by Olivon and collaborator in 2019. MS/MS data converted to mzXML format were processed using mzmine 2 v2.53 53. All parameters were presented in Table S2.

## Conflict of interest

The authors declare that they have no conflict of interest.

## Supporting information


**Fig. S1.** Tryptophan relative quantification in un‐inoculated broth and agar media. Evaluation of the efficiency of the extraction method for recovering secondary metabolites embedded in agar medium was carried out thanks to tryptophan (Trp). Tryptophan is a precursor of several secondary metabolite biosynthesis pathways in bacteria; in un‐inoculated media, it could be used for method validation. Tryptophan was detected in the first third of the chromatogram; it has a median polarity close to that of numerous metabolites. Its relative quantification in un‐inoculated MM broth and MM agar does not show significant difference (*P*‐value = 0.21, Wilcoxon test), which indicates comparable efficiency of the extraction method for metabolites of median polarity between the 2 conditions.

**Fig. S2.** Chemical structures of some secondary metabolites from the studied plant‐associated *Pseudomonas* strains. Transformation of phenazine‐1‐carboxylic acid into different derivatives thanks to genes *phzO* in JV395B and* phzH* in JV497 (A). Chemical structures of 3‐OH‐C_6_‐HSL, 3,4‐Dihydro‐5‐methyl‐4‐alkenyl(C13:1)‐2‐H‐pyrrole and PDTC (pyridine‐2,6‐thiocarboxylic acid) derivatives (B).

**Table S1.** List of 8 chemical standards used in UHPLC‐DAD‐qTOF analyses and parameter settings used for metabolomics data processing.

**Table S2.** MZmine 2 data‐preprocessing parameters for molecular networking.
