# Supplementary material for: Secondary metabolites from plant‐associated Pseudomonas are overproduced in biofilm
Source: Microb Biotechnol. 2020 Aug 9;13(5):1562–80. doi: 10.1111/1751-7915.13598 (PMC7415375; doi:10.1111/1751-7915.13598)
Supplement: Supplementary file 1 — Fig. S1. Tryptophan relative quantification in un‐inoculated broth and agar media. Evaluation of the efficiency of the extraction method for recovering secondary metabolites embedded in agar medium was carried out thanks to tryptophan (Trp). Tryptophan is a precursor of several secondary metabolite biosynthesis pathways in bacteria; in un‐inoculated media, it could be used for method validation. Tryptophan was detected in the first third of the chromatogram; it has a median polarity close to that of numerous metabolites. Its relative quantification in un‐inoculated MM broth and MM agar does not show significant difference (P‐value = 0.21, Wilcoxon test), which indicates comparable efficiency of the extraction method for metabolites of median polarity between the 2 conditions. [file MBT2-13-1562-s001.docx]

**Figure S1**. **Tryptophan relative quantification in un-inoculated broth and agar media.** Evaluation of the efficiency of the extraction method for recovering secondary metabolites embedded in agar medium was carried out thanks to tryptophan (Trp). Tryptophan is a precursor of several secondary metabolite biosynthesis pathways in bacteria; in un-inoculated media, it could be used for method validation. Tryptophan was detected in the first third of the chromatogram; it has a median polarity close to that of numerous metabolites. Its relative quantification in un-inoculated MM broth and MM agar does not show significant difference (*P*-value = 0.21, Wilcoxon test), which indicates comparable efficiency of the extraction method for metabolites of median polarity between the 2 conditions.
