# Supplementary material for: Secondary metabolites from plant‐associated Pseudomonas are overproduced in biofilm
Source: Microb Biotechnol. 2020 Aug 9;13(5):1562–80. doi: 10.1111/1751-7915.13598 (PMC7415375; doi:10.1111/1751-7915.13598)
Supplement: Supplementary file 2 — Fig. S2. Chemical structures of some secondary metabolites from the studied plant‐associated Pseudomonas strains. Transformation of phenazine‐1‐carboxylic acid into different derivatives thanks to genes phzO in JV395B and phzH in JV497 (A). Chemical structures of 3‐OH‐C6‐HSL, 3,4‐Dihydro‐5‐methyl‐4‐alkenyl(C13:1)‐2‐H‐pyrrole and PDTC (pyridine‐2,6‐thiocarboxylic acid) derivatives (B). [file MBT2-13-1562-s002.docx]

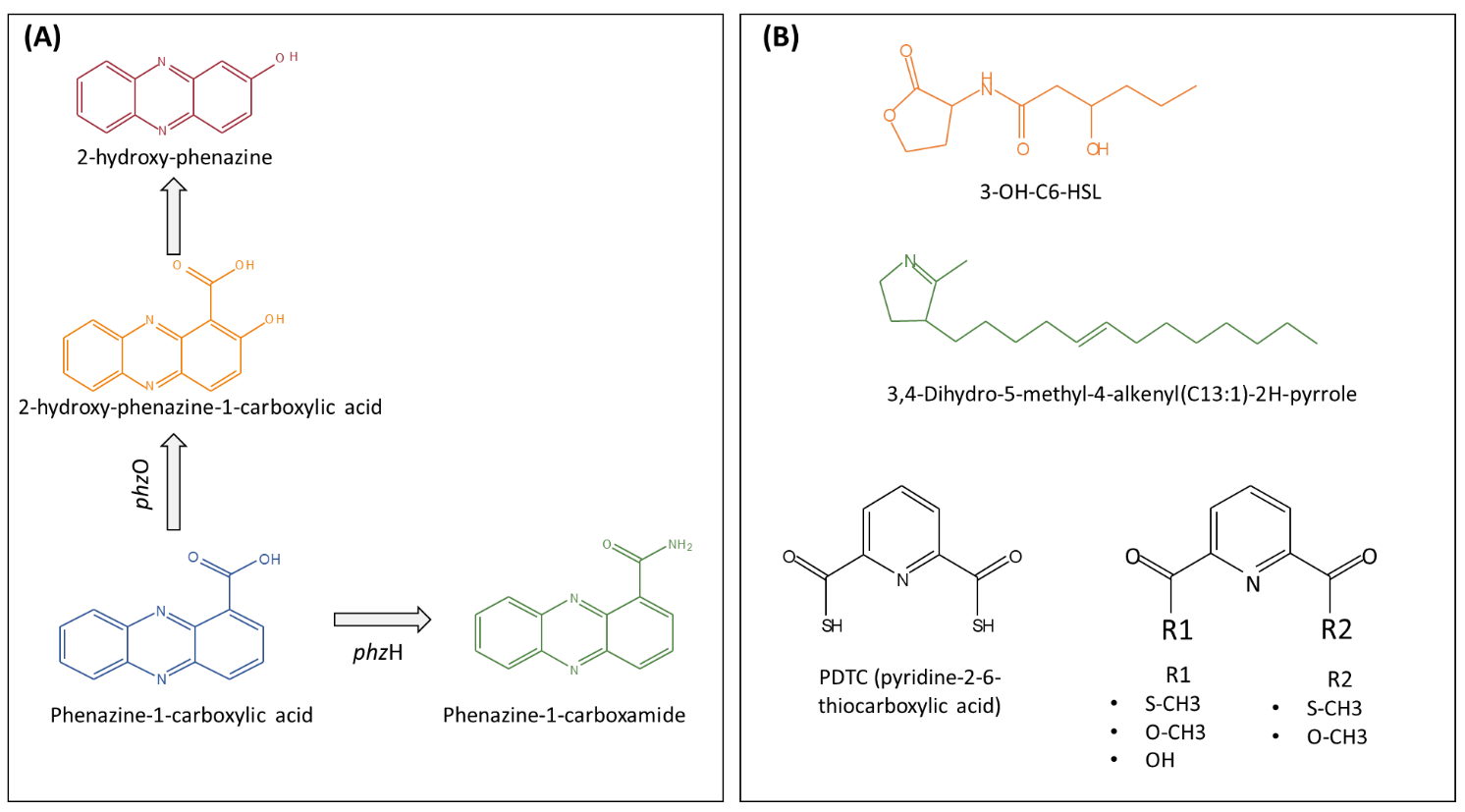
**Figure S2. Chemical structures of some secondary metabolites from the studied plant-associated *Pseudomonas* strains.** Transformation of phenazine-1-carboxylic acid into different derivatives thanks to genes *phzO* in JV395B and *phzH* in JV497 **(A)**. Chemical structures of 3-OH-C_6_-HSL, 3,4-Dihydro-5-methyl-4-alkenyl(C13:1)-2-H-pyrrole and PDTC (pyridine-2,6-thiocarboxylic acid) derivatives **(B)**.
