# Supplementary material for: Secondary metabolites from plant‐associated Pseudomonas are overproduced in biofilm
Source: Microb Biotechnol. 2020 Aug 9;13(5):1562–80. doi: 10.1111/1751-7915.13598 (PMC7415375; doi:10.1111/1751-7915.13598)
Supplement: Supplementary file 3 — Table S1. List of 8 chemical standards used in UHPLC‐DAD‐qTOF analyses and parameter settings used for metabolomics data processing. [file MBT2-13-1562-s003.docx]

**Table S1. List of 8 chemical standards used in UHPLC-DAD-qTOF analyses and parameter settings used for metabolomics data processing.**

| **Chemical Standards** | | | | |
| --- | --- | --- | --- | --- |
| **N°** | **Name** | **Molecular formula** | **Origin** | **Purity** |
| 1 | Monoacetylphloroglucinol | C_8_H_8_O_4_ | Cayman Chemical | ≥98% |
| 2 | 2,4-diacetylphloroglucinol | C_10_H_10_O_5_ | Toronto Research Chemicals Inc. | ≥98% |
| 3 | Pyrrolnitrin | C_10_H_6_Cl_2_N_2_O_2_ | SIGMA Chemical Co. | ≥98% |
| 4 | Phenazine-1-carboxylic acid | C_13_H_8_N_2_O_2_ | Toronto Research Chemicals Inc. | ≥98% |
| 5 | 3-OH-C6-HSL | C_10_H_17_NO_4_ | SIGMA Chemical Co. | ≥98% |
| 6 | Dimethyl 2,6-pyridinedicarboxylate | C_9_H_9_NO_4_ | SIGMA Chemical Co. | ≥98% |
| 7 | Indole-acetic-acid | C_10_H_9_NO_2_ | SIGMA Chemical Co. | ≥98% |
| 8 | Tryptophan | C_11_H_12_N_2_O_2_ | SIGMA Chemical Co. | ≥98% |

| **Parameter settings used for metabolomics data processing** | | | |
| --- | --- | --- | --- |
| **Step** | **Method** | **Parameters** | **Values** |
| Peak detection (*Peakpicking*)^a^ | centWave | ppm | 10 |
|  |  | mzdiff | 0.05 |
|  |  | prefilter | 3, 10000 |
|  |  | snthresh | 10 |
|  |  | peakwidth | 4, 15 |
|  |  | noise | 12000 |
| Peak grouping (*Group*)^a^ | density | bw | 5 |
|  |  | mzwid | 0.25 |
|  |  | minfrac | 0.05 |
| FillPeaks | chrom |  |  |

^a^ data processed with xcms R-package on collaborative Galaxy platform “Workflow4metabolomics” version 3.3

**Table S2. MZmine 2 data-preprocessing parameters for molecular networking**

| **Steps** | **Methods** | **Parameters** | **Values** |
| --- | --- | --- | --- |
| **Mass detection** |  | Noise level MS1 | 0 |
|  |  | Noise level MS2 | 0 |
| **Chromatogram builder** | ADAP | Minimum group size of scan | 4 |
|  |  | Group intensity threshold | 3000 |
|  |  | Minimum highest intensity | 4000 |
|  |  | m/z tolerance | 0.005 (20 ppm) |
| **Deconvolution** | ADAP Wavelets algorithm | S/N threshold | 8 |
|  |  | Minimum feature height | 4000 |
|  |  | Coefficient/area threshold | 20 |
|  |  | Peak duration range | 0.05 – 1 min |
|  |  | T_R_ wavelet range | 0.01 – 0.07 min |
| **MS2 scans paired** |  | m/z tolerance | 0.02 Da |
|  |  | t_R_ tolerance | 0.3 min |
| **Isotopologue grouping** | Isotopic peak grouper algorithm | m/z tolerance | 0.005 (20 ppm) |
|  |  | t_R_ tolerance | 0.2 min |
| **Filtering** | Feature list rows filter | Retention time range | 1 – 14 min |
|  |  | **Keep only peaks with MS2 scan** | |
| **Peak alignment** | Join aligner module | m/z tolerance | 0.005 (20 ppm) |
|  |  | Weight for m/z | 2 |
|  |  | t_R_ tolerance | 0.5 min |
|  |  | Weight for t_R_ | 1 |
| **Gap filled** |  | m/z tolerance | 0.005 (20 ppm) |
|  |  | t_R_ tolerance | 0.5 min |
