# Supplementary material for: Secondary metabolites from plant‐associated Pseudomonas are overproduced in biofilm
Source: Microb Biotechnol. 2020 Aug 9;13(5):1562–80. doi: 10.1111/1751-7915.13598 (PMC7415375; doi:10.1111/1751-7915.13598)
Supplement: Supplementary file 4 — Table S2. MZmine 2 data‐preprocessing parameters for molecular networking. [file MBT2-13-1562-s004.docx]

**Table S2. MZmine 2 data-preprocessing parameters for molecular networking**

| **Steps** | **Methods** | **Parameters** | **Values** |
| --- | --- | --- | --- |
| **Mass detection** |  | Noise level MS1 | 0 |
|  |  | Noise level MS2 | 0 |
| **Chromatogram builder** | ADAP | Minimum group size of scan | 4 |
|  |  | Group intensity threshold | 3000 |
|  |  | Minimum highest intensity | 4000 |
|  |  | m/z tolerance | 0.005 (20 ppm) |
| **Deconvolution** | ADAP Wavelets algorithm | S/N threshold | 8 |
|  |  | Minimum feature height | 4000 |
|  |  | Coefficient/area threshold | 20 |
|  |  | Peak duration range | 0.05 – 1 min |
|  |  | T_R_ wavelet range | 0.01 – 0.07 min |
| **MS2 scans paired** |  | m/z tolerance | 0.02 Da |
|  |  | t_R_ tolerance | 0.3 min |
| **Isotopologue grouping** | Isotopic peak grouper algorithm | m/z tolerance | 0.005 (20 ppm) |
|  |  | t_R_ tolerance | 0.2 min |
| **Filtering** | Feature list rows filter | Retention time range | 1 – 14 min |
|  |  | **Keep only peaks with MS2 scan** | |
| **Peak alignment** | Join aligner module | m/z tolerance | 0.005 (20 ppm) |
|  |  | Weight for m/z | 2 |
|  |  | t_R_ tolerance | 0.5 min |
|  |  | Weight for t_R_ | 1 |
| **Gap filled** |  | m/z tolerance | 0.005 (20 ppm) |
|  |  | t_R_ tolerance | 0.5 min |
